# Novel *NARS2* variants in a patient with early-onset status epilepticus: case study and literature review

**DOI:** 10.1186/s12887-024-04553-0

**Published:** 2024-02-03

**Authors:** Nuo Yang, Limin Chen, Yanfeng Zhang, Xuemei Wu, Yunpeng Hao, Fan Yang, Zuozhen Yang, Jianmin Liang

**Affiliations:** 1 Department of Pediatric Neurology, First hospital of Jilin University, Changchun, 130021 China; 2 Jilin Provincial Key Laboratory of Pediatric Neurology, Changchun, 130021 China; 3 Cipher Gene LLC, Beijing, 100089 China

**Keywords:** NARS2 protein, Global developmental delay, Hyperlactatemia, Epilepsy, Myocardial creatine kinase

## Abstract

**Background:**

NARS2 as a member of aminoacyl-tRNA synthetases was necessary to covalently join a specific tRNA to its cognate amino acid. Biallelic variants in *NARS2* were reported with disorders such as Leigh syndrome, deafness, epilepsy, and severe myopathy.

**Case presentation:**

Detailed clinical phenotypes were collected and the *NARS2* variants were discovered by whole exome sequencing and verified by Sanger sequencing. Additionally, 3D protein structure visualization was performed by UCSF Chimera. The proband in our study had early-onset status epilepticus with abnormal EEG and MRI results. She also performed global developmental delay (GDD) and myocardial dysfunction. Next-generation sequencing (NGS) and Sanger sequencing revealed compound heterozygous missense variants [NM_024678.6:exon14: c.1352G > A(p.Arg451His); c.707T > C(p.Phe236Ser)] of the NARS2 gene. The proband develops refractory epilepsy with GDD and hyperlactatemia. Unfortunately, she finally died for status seizures two months later.

**Conclusion:**

We discovered two novel missense variants of *NARS2* in a patient with early-onset status epilepticus and myocardial dysfunction. The NGS enables the patient to be clearly diagnosed as combined oxidative phosphorylation deficiency 24 (COXPD24, OMIM:616,239), and our findings expands the spectrum of gene variants in COXPD24.

**Supplementary Information:**

The online version contains supplementary material available at 10.1186/s12887-024-04553-0.

## Introduction

Asparaginyl-tRNA synthetase 2 (Asn-RS) encoded by *NARS2* is a member of the class II family of aminoacyl-tRNA synthetases (aaRSs) which play a crucial role in biosynthesis by catalyzing the ligation of asparagine to tRNA molecules [1]. This protein was first identified in 2005 [2] and contains 477 amino acids. It is expressed ubiquitously throughout the body both in humans and mice [1]. Moreover, it is expected to function as a dimer [2].

Pathogenic variants in *NARS2* have been subsequently identified [3, 4] and were correlated to the combined oxidative phosphorylation deficiency 24 (COXPD24) (OMIM: 616,239) which is an autosomal recessive mitochondrial disorder that exhibits pleiotropic phenotypes. It is associated with visual and hearing abnormalities, myopathy, neurodevelopmental disorder, and mitochondrial dysfunction [5–9].

Here we present a further patient from a non-consanguineous family with an infantile-onset neurodegenerative disorder characterized by status epilepticus, increased serum lactic acid, and abnormal brain structure. The novel compound heterozygous variants in *NARS2* [NM_024678.6: c.1352G > A(p.Arg451His); c.707T > C(p.Phe236Ser)] were identified by whole exome sequencing (WES). And our findings expand the genotype spectrum of COXPD24.

## Methods

### Patient

Patient with early-onset status epilepticus have been confirmed at the First hospital of Jilin University. Informed consent was provided from the families contained according to institutional guidelines. Ethics approval has been obtained by the human ethics committees of Bethune First Hospital of Jilin University. The clinical, laboratory examinations, and genetic tests were obtained for the patients.

### WES

The genomic DNA isolated from the peripheral blood of our patient, her parents and brother. Exome captures were performed using the IDT xGen Exome Research Panel with paired-end read sequences generated on NovaSeq 6000 sequencing. Sequences were aligned to Human reference genome GRCh38/hg38 using the Burrows-Wheeler Aligner (BWA) [10]. The variants were then annotated through AnnoVar [11] and evaluated according to allele frequencies, pathogenicity prediction, and protein function. The pathogenicity of variants were predicted in silico for missense variants (SIFT, PolyPhen2, LRT, MutationTaster, FATHMM, CADD, REVEL) and for splice site variants (MaxEntScan, NNSplice, dbscSNV) [12]. Variants with minor allele frequency < 0.005 were selected, and were classified according to inheritance pattern. Candidate variants were finally screened according to the American College of Medical Genetics and Genomics (ACMG) [13] classification guidelines and clinical phenotypes.

The criteria for variant filtering were as follows:

1. Variants located in exon and splicing (± 20 bp) region and minor allele frequency < 0.005 for genome aggregation database (gnomAD) exome_popmax, gnomAD_genome_popmax, gnomAD3_genome_AF_ Popmax, and etc. were selected.
2. Missense variations predicted harmful by most commonly used software will be adopted.
3. Then variants were classified according to inheritance pattern: de novo variants, autosomal recessive (AR) inheritance of homozygous variants, AR inheritance of compound heterozygous variants, X-linked inheritance (Supplementary Table 1).

Pathogenic variants related to clinical phenotypes will further be verified by Sanger sequencing. Primers were designed with Primer3 software [14]. Polymerase chain reaction (PCR) amplified products were purified and then sequenced with BigDye v3.1 (Applied Biosystems).

### 3D protein structure modeling

Molecular modeling analysis was performed to show the variations in protein structure. The homology models in the NARS2 protein based on the crystal structure of the Elizabethkingia Asparagine-tRNA ligase were predicted by the Swiss-Model program [15]. The human NARS2 model was downloaded in the AlphaFold dataset [16]. UCSF Chimera software was used to visualize the structures in dimmers and monomers [17].

## Results

### Case presentation

The patient was the second child of healthy non-consanguineous parents. This patient was born on an uneventful full-term cesarean delivery with a birth weight of 3.55 kg. However, the global developmental delay (GDD) was found in our patient with difficulty to head control, roll over, eyes following objects, and feeding in her three-month-old. Furthermore, her weight gains slowly after birth, weighing only 5.5 kg at 3 months old.

**Fig. 1:**
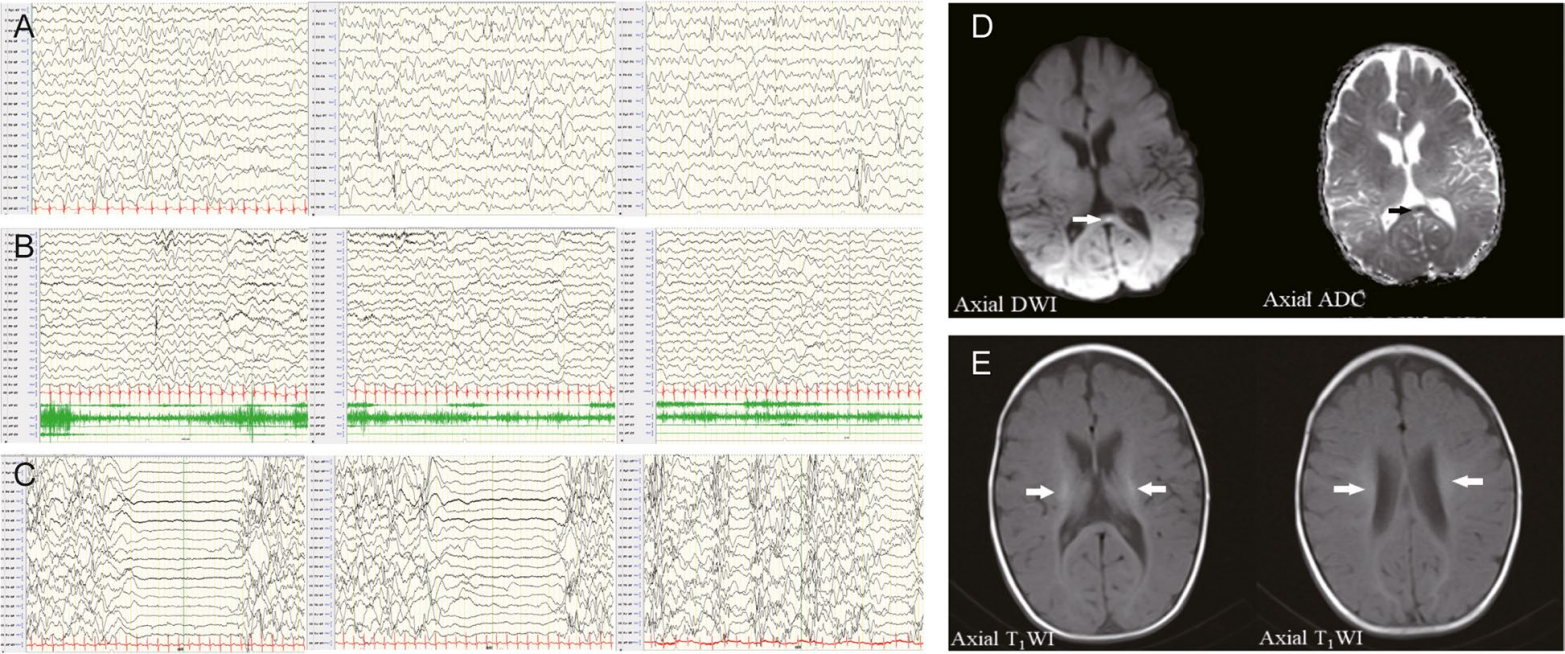
EEG and MRI results in patient with *NARS2* heterozygous variant. **A** EEG results showed multiple foci-spikes, spike waves, and sharp waves when patient was 3 months and 20 days old. **B** The patient exhibited upward rolling of the eyes and salivation, while the EEG shows synchronization with low to medium amplitude fast wave rhythm in the left frontotemporal. **C** The EEG presented highly irregular burst suppression patterns when patient was 4 months and 16 days old. **D** At the age of 3 months and 12 days, the diffusion-weighted image (DWI) revealed a small and slightly high signal shadow in the corpus callosum. while an apparent diffusion coefficient map (ADC) showed a slightly low signal intensity. **E** At the age of 4 months and 15 days, the T1-weighted image showed abnormalities in bilateral symmetry signal, and decreased white matter in bilateral cerebral

She was admitted to our hospital due to intermittent fever, seizures, eyes upward rolling and salivation when she was 3 months old. She was initially diagnosed with epilepsy and developmental delay for the abnormal electroencephalograph (EEG) and Magnetic Resonance Imaging (MRI) results. EEG showed the background rhythm slowed down and mixture multiple foci-spikes, spike waves, and sharp waves (Fig. 1A). There were frequent focal subclinical or clinical seizures arising from the left frontotemporal (Fig. 1B). An abnormal signal in the splenium of the corpus callosum was shown in MRI (Fig. 1D). Her intermittent fever was gradually controlled through anti-infection treatment. However, the status seizures failed to remission for continuous adjustment the types and dosages of antiepileptic drug.

She also had a myocardial dysfunction with elevated myocardial creatine kinase (CK-MB 46.0U/L, ref: 0-25U/L) and B-type natriuretic peptide precursor (PRO-LPBN 209.0pg/ml, ref: 0-125pg/ml). Sodium creatine phosphate was given as nutritional myocardial therapy. Furthermore, increased serum lactic acid (5.8mmol/L, ref: 0.5-2.2mmol/L) was suspected for mitochondrial genetic disease. The brainstem auditory evoked potentials (BAEP) showed bilateral suspicious peripheral damage, combined with central damage suggesting hearing impairment. EEG and MRI were rechecked after 1 week in hospital. EEG present highly irregular with burst suppression patterns (Fig. 1C). White matter volume was reduced and bilateral symmetry signal abnormalities were shown in repeat MRI (Fig. 1E). She was admitted to the intensive care unit two times for status seizures. Twenty-six days after admission, her seizures were still frequent. Her breathing and swallowing decreased, her heart rate, blood oxygen, and various vital signs were not stable. She was discharged due to her parents’ strong request. She was still suffered from feeding difficulties and with breathing difficulty. Unfortunately, she passed away (8-month-old) at the local hospital with multiple organ failure and malnutrition 3 months later.

### Identification of *NARS2* variations by WES

Trio WES was subsequently performed to further investigate the etiology for the patient from a non-close relative’s family. Variants were filtered by the minor allele frequency (MAF), related phenotype and predicted damage. We listed variants as candidate pathogenic genes (Supplementary Table 1), some of which were excluded because they only explained part of the patient’s phenotype, or the inheritance pattern did not match. Two novel compound heterozygotes in *NARS2* [NM_024678.6: c.1352G > A (p.Arg451His); c.707T > C (p.Phe236Ser)] were identified. These two variants have not been included in public data such as the gnom AD (https://gnomad.broadinstitute.org/gene/ENSG00000134440?dataset=gnomad_r2_1) [18, 19], and variant c.1352G > A (p.Arg451His) has a very low MAF with 0.00003184 in gnom AD_genome_ALL (Table 1). Additionally, 107 single nucleotide variations in *NARS2* were recorded so far in ClinVar https://www.ncbi.nlm.nih.gov/clinvar/?term=NARS2%5Bgene%5D, and neither of the two variants in our patient were included. And both variants in our patient were predicted being damage by several prediction software (Table 1). It seems that variants in *NARS2* are rare both in the disease group (Clinvar) or general population (gnom AD), and the pathogenicity needs to be further investigated.

**Table 1:** Variants information in our patient

Gene	Variant	Inheritance	MAF			Variants hazard prediction		
			ExAc	gnomAD_genome_ALL	1000 genome	SIFT	Polyphen2_HDIV	Mutation Taster
*NARS2*	c.1352G > A, p.Arg451His	AR	NE	0.00003184	NE	Deleterious	Probably damaging	Disease_causing
	c.707T > C, p.Phe236Ser		NE	NE	NE	Deleterious	Probably damaging	Disease_causing

Transcript: NM_024678.6

*AR* Autosomal recessive inheritance, *MAF* Minor allele frequency, *NE* Not exist

**Fig. 2:**
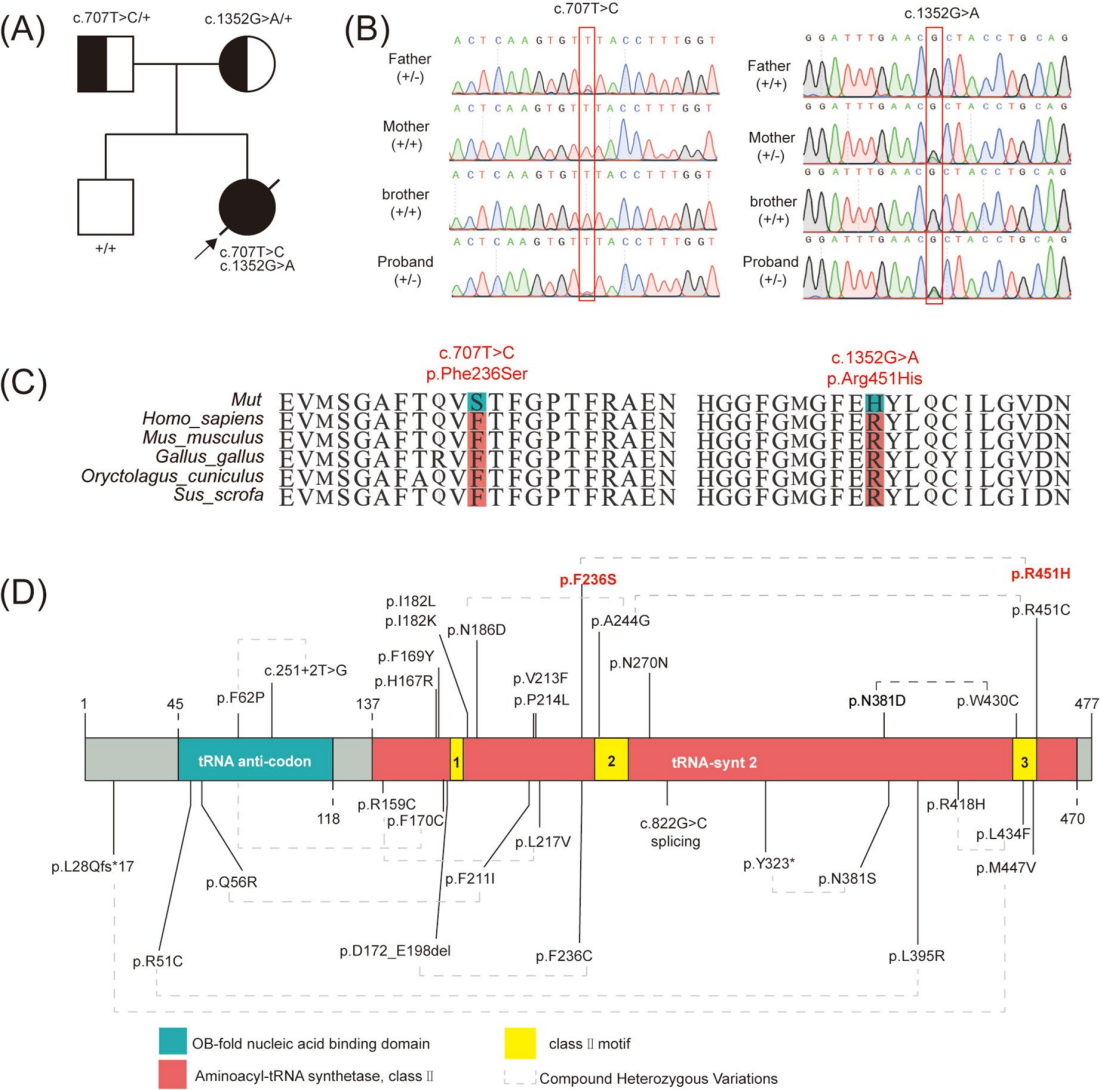
The variant information of *NARS2*. **A** The pedigree of this family. The proband affected with status seizures is indicated by black filled symbols and arrows. The parents who carried variants are displayed by symbols with black dots in the center. **B** Sanger sequencing of this family showed compound heterozygous variants c.70T > c, C.1352G > A (red box) in the proband were inherited from her parents respectively. **C** Variants of *NARS2* in our patient located highly conserved areas based on the comparison performed among multiple species. **D** Domain structure and modeling of the known *NARS2* variations in previous studies. The NARS2 protein contains an OB-fold nucleic acid binding domain (green) and an aminoacyl-tRNA synthetase domain (red). Three conservative motifs were shown in the structure (yellow). Variants in our study were highlighted in red font. All the compound heterozygous variations were linked by a dashed gray line

The heterozygous variants in the patient were confirmed by Sanger sequencing and they were inherited from her parents (Fig. 2A-B). The variant c.707T > C was inherited from her father, and another variant c.1352G > A was inherited from her mother (Fig. 2B). All these two variants changed the amino acids were conserved in multiple species (Fig. 2C), may indicating their important functions. Studies of patients with *NARS2* variations showed variable clinical phenotypes (Table 2). They may also be associated with additional complications as various degrees of intellectual disability, visual, hearing impairment, and developmental delay. NARS2 gene variations were identified in patients with autosomal recessive deafness and COXPD24, and most of them were missense. A schematic diagram of NARS2 variations was shown in Fig. 2D.

**Table 2:** *NARS2 *variants and associated phenotypes in previous studies

**Author/year **	**Onset age**	**Survivaloutcome** **/age**	**Diagnosis**	**Close relative**	**Zygote type**	**Variation**	**Variant type**	**Case No.**
Vanlander et al. 2015 [4]	Not mention	Alive 34y	Combined oxidative phosphorylation deficiency 24	Con	HMZ	c.822G>C; p.Q274H; Chr11(GRCh37):g.78204109C>G	Missense	1
	Childhood	Alive 26y	Combined oxidative phosphorylation deficiency 24					2
Sofou et al. 2015 [3]	Perinatal	Deceased 16y	Alpers syndrome	Non-con	HMZ	c.641C>T; p.Pro214Leu	Missense	3
Simon et al. 2015 [1]	Infantile	Deceased 15m	Leigh Syndrome	Non-con	HTZ	c.969T>A; p.Tyr323*c.1142A>G; p.Asn381Ser	Truncation Missense	4
	Infantile	Deceased 6m	Leigh Syndrome					5
	Not mention	Alive 45y	Autosomal recessive deafness	Con	HMZ	c.637G>T; p.Val213Phe	Missense	6
Mizuguchi et al. 2017 [5]	8m	Alive 8y	Infantile-onset neurodegenerative disorder	Not mention	HTZ	c.707T>G; p.Phe236Cysc.594+1G>A; p.Asp172_Glu198del	Missense	7
	10m	Alive 1y	Infantile-onsetneurodegenerative disorder				Missense	8
	8m	Alive 2y	Infantile-onset neurodegenerative disorder	Not mention	HTZ	c.151C>T; p.Arg51Cysc.1184T>G; p.Leu395Arg	Missense	9
	4m	Alive 4y	Infantile- onsetneurodegenerative disorder	Not mention	HMZ	c.500A>G; p.His167Arg	Missense	10
Seaver et al. 2018 [6]	3m	Deceased 6m	Combined oxidative phosphorylation deficiency 24	Non-con	HTZ	c.167A>G; p.Gln56Argc.631T>A; p.Phe211Ile	Missense	11
	4m	Deceased 9m	Combined oxidative phosphorylation deficiency 24					12
Lee et al. 2020 [20]	Not mention	Not mention	Leigh syndrome	Non-con	HTZ	c.731C>G; p.Ala244Glyc.1351C>T; p.Arg451Cys	Missense	13
Palombo et al. 2020 [21]	Perinatal	Alive 22y	Reversible COX deficiency	Con	HMZ	c.270C> T; p.Asn90Asn	Synonymous	14
Sofou et al. 2021 [7]	Perinatal	Deceased 6y	Alpers syndrome	Non-con	HMZ	c.641C>T; p.Pro214Leu	Missense	15
	5m	Alive 25y	Alpers /Leigh syndrome					16
Vafaee et al. 2021 [9]	12m	Alive 17y	Combined oxidative phosphorylation deficiency 24	Con	HMZ	c.545T>A; p.Ile182Lys	Missense	17
	6m	Alive 28m						18
Štěrbová et al. 2021 [8]	3.5m	Deceased 14m	Fatal refractory status epilepticus	Non-con	HTZ	c.83_84del; p.Leu28Glnfs*17c.1339A>G; p.Met447Val	Truncation missense	19
Zhang et al. 2022 [22]	3m	Deceased 6m	Combined oxidative phosphorylation deficiency 24	Non-con	HTZ	c.1141A>G; p.Asn381Aspc.1290G>C; p.Trp430Cys	Missense	20
Yagasaki et al. 2022 [23]	3m	Alive 3y	DD, Epilepsy, and neonatal diabetes (DEND) syndrome	Non-con	HTZ	c.475C>T; p.Arg159Cysc.649T>G; p.Leu217Val	Missense	21
	Infantile	Alive 1y						22
Yang et al. 2022 [24]	Infantile	Alive 1y	Leigh syndrome	Non-con	HTZ	c.1253G>A; p.Arg418Hisc.1300C>T; p.Leu434Phe	Missense	23
Tanaka R et al. 2022 [25]	Infantile	Alive	Leigh Syndrome	Non-con	HTZ	c.556 A>G; p.Asn186Aspc.731 C>G; p.Ala244Gly	Missense	24
Al-Sharif et al. 2022 [26]	14m	Alive 3y	Bilateral Nonsyndromic Sensorineural Hearing Loss	Con	HTZ	c.506T>A; p.Phe169Tyr	Missense	25
Cokyaman T et al. 2022 [27]	Neonatal period	Alive 14m	Type 1 diabetes mellitus	Con	HMZ	c.500 A>G; p.H167R	Missense	26
Hu W et al. 2022 [28]	2m	Deceased 11m	Intractable refractory epilepsia partialis continua; DD	Non-con	HTZ	c.185T > C; p.Leu62Pro and c.251 + 2T > G	Splicing	27
	5m	Alive 5m	Intractable refractory epilepsia partialis continua; DD	Non-con	HTZ	c.185T > C; p.Leu62Pro and c.509T > G/p.Phe170Cys	Splicing	28
Our study	3m	Deceased 8m	Epilepsy; DD	Non-con	HTZ	c.1352G>A; p.Arg451Hisc.707T>C; p.Phe236Ser	Missense	29

**Author/year **	**Gender**	**Myopathy phenotype**	**Visual phenotype**	**Hearing phenotype**	**Neurodevelopmental disorder**	**EEG**	**MRI/CT**	**Lactate**
Vanlander et al. 2015 [4]	Female	Proximal muscle weakness; Severe amyotrophy; Paresis of facial muscles	Not mention	Not mention	Not mention	No t mention	Normal	Normal
	Male	No signs of myopathy	Not mention	Not mention	Mild ID	Epilepsy	Normal	Not mention
Sofou et al. 2015 [3]	Male	Hypotonia; Spastic tetraparesis	Optic atrophy and nystagmus, and later developed cortical visual impairment leading to blindness	Not mention	Severe ID; Psychomotor regression Generalized seizures of multiple types	Bilateral synchronous spikes and polyspikes , mainly in the posterior regions of the hemispheres, with generally depressed background activity	Supratentorial atrophy of the cerebral cortex; Complete agenesis of the corpus callosum; Hypomyelination of the white matter	Elevated
Simon et al. 2015 [1]	Male	Not mention	Not mention	Hearing abnormal	Complex partial seizures	Status epilepticus	Multiple areas of hyperintensive T2-weighted and Fluid- attenuated inversion recovery (FLAIR) signal within periventricular white matter and posterior corona radiata with extension into the posterior limbs of the internal capsule. There was also a hyperintensive signal in the thalami and dentate nuclei	Elevated
	Male	Not mention	Not mention	Hearing abnormal	Continuous left hemispheric focal seizures	Continuous left hemispheric focal seizures	Diffusion in the left basal ganglia, and external capsule junction as well as the left frontal lobe in cortical distribution	Normal
	Female	Not mention	No hypotonia	Hearing abnormal	No seizure history	Not mention	Not determined	Not mention
Mizuguchi et al. 2017 [5]	Male	Flaccid quadriplegia	Optic nerve atrophy	Hearing impairment	Severe ID; Microcephaly Psychomotor regression	Diffuse spikes and slow-wave complexes	Diffuse brain atrophy	Elevated
	Female	Flaccid quadriplegia	Not mention	Severe bilateral hearing impairment	ID; Microcephaly Developmental regression	Multifocal spikes	Normal	Elevated
	Female	Muscle weakness in her all extremities and pharynx	Not mention	Severe bilateral hearing impairment	ID; Hemi-convulsive status epilepticus	Frequent spikes and wave complexes in the left occipital area while awake, and modified hypsarrhythmia during sleep	Diffuse atrophic changes in the left cerebrum	Elevated
	Male	Spastic quadriplegia	Not mention	Severe bilateral hearing impairment	ID; Microcephaly Psychomotor regression	Burst suppression pattern	Cerebral atrophy with extended vacuolization of the periventricular white matter, basal ganglia, corpus callosum and cerebellum	Elevated
Seaver et al. 2018 [6]	Male	Nonspecific myopathic changes	Not mention	Not mention	Focal status epilepticus	Frequent epileptiform discharges over the left centrotemporal leads	Progressive diffuse cerebral volume loss and increased subdural effusions	Normal
	Male	Not mention	Not mention	Hearing abnormal	Focal status epilepticus	Seizures originating from the left centroparietal region	Progressive white matter T2 hyperintensity, volume loss, bifrontal subdural effusions, and widespread cerebral restricted diffusion	Normal
Lee et al. 2020 [20]	Not mention	Not mention	Not mention	Not mention	Not mention	Not mention	Not mention	Not mention
Palombo et al. 2020 [21]	Not mention	Hypotonia	Not mention	Hearing loss	Psychomotor regression	Abnormal	Asymmetry of the hippocampus	Elevated
Sofou et al. 2021 [7]	Female	Spastic quadriplegia	Cortical blindness	Hearing abnormal	Profound DD; Treatment-resistant epilepsy	Multifocal epileptiform activity and slowing of the background activity	Global cerebral atrophy	Elevated
	Male	Spastic quadriplegia	Not mention	Sensorineural hearing impairment	Profound DD; Treatment-resistant epilepsy	Generalized tonic- clonic and myoclonic seizures	Basal ganglia signal abnormalities	Elevated
Vafaee et al. 2021 [9]	Female	Normal	Not mention	Hearing abnormal	Generalized tonic-colonic seizures; Developmental regressed; Mild ID	Bilateral synchronous spike and polyspike waves mainly in the posterior part of the brain	Normal	Not mention
	Female	Normal	Not mention	Hearing abnormal	Generalized tonic-colonic seizures; Developmental regressed	Bilateral synchronous spike and polyspike waves mainly in the posterior part of the brain	Normal	Not mention
Štěrbová et al. 2021 [8]	Male	Subtle atrophy of the muscle fibres	Not mention	Not mention	Focal status epilepticus	Bilateral clonic and myoclonic seizures	Progressive cortical and periventricular brain atrophy	Normal
Zhang et al. 2022 [22]	Male	Muscle weakness and hypotonia	Not mention	Severe bilateral hearing impairment	Early onset generalized epilepsy; DD	Rhythmic slow waves mixed with irregular spikes, as well as sharp slow waves in the central, parietal, and temporal regions	Normal	Elevated
Yagasaki et al. 2022 [23]	Female	Muscle weakness and hypotonia	Not mention	Hearing loss	Severe DD; Treatment-resistant epilepsy	Multifocal epileptiform activity and slowing of background activity	Lightly atrophic at the frontal lobe	Elevated
	Male	Muscle weakness and hypotonia	Not mention	Hearing loss	Severe DD; Treatment-resistant epilepsy	Multifocal epileptiform activity and slowing of background activity	Atrophy	Elevated
Yang et al. 2022 [24]	Male	Muscle weakness and hypotonia	Not mention	Not mention	DD; Myoclonic seizures	Not mention	Symmetric, bilateral lesions of hyperintense T2-weighted and FLAIR signal in bilateral basal ganglia and lenticular nuclei	Not mention
Tanaka R. 2022 [25]	Female	Severe muscular weakness	Not mention	Hearing abnormal	Generalized tonic and myoclonic seizures; Developmental regression	Generalized spike-waves	Normal	Normal
Al-Sharif et al. 2022 [26]	Male	Normal	Normal	Bilateral Hearing loss	Language development was delayed	Not mention	Normal	Not mention
Cokyaman T et al. 2022 [27]	Female	Hypotonia	Normal	Hearing loss	Refractory myoclonic epilepsy; severe DD	Spike discharges were detected with irregularity and slowdown in the occipital back-ground rhythm	Subdural hemorrhagic hygroma	Normal
Hu W et al. 2022 [28]	Female	Hypotonia	Not mention	Hearing abnormal	Intractable refractory epilepsia partialis continua; DD	Background rhythm slowed down, sharp waves in the central, top, occipital, and midline electrodes	Abnormal signal shadows in the internal and external capsules and the left rahippocampalgyrus.	Not mention
	Female	Hypotonia	Not mention	Hearing abnormal	Intractable refractory epilepsia partialis continua; DD	In the background of a diffuse rhythm, the top, occipital, and middle and posterior temporal electrodes(mostly right side) showed a low-to-medium amplitude spike wave rhythm, affecting the central region	The bilateral cerebral hemisphere sulcus fissures had widened and deepened; The bilateral frontotemporal extra cerebral spaces had widened slightly; The bilateral lateral ventricles had enlarged slightly ; Diffusion-weighted imaging showed slightly high signal intensities at the partial cortex of the cerebral hemisphere and the left hippocampus	Elevated
Our study	Female	Myocardial dysfunction	Not mention	Hearing abnormal	Epilepsy; DD	Multiple foci-spikes, spike waves, and sharp waves, focal subclinical or clinical seizures	Bilateral symmetry signal abnormalities	Elevated

*Con* Consanguineous parents, *Non-con *Non-consanguineous parents, *m *Month, *y *year, *DD *Developmental delay, *ID *Intellectual disability, *HMZ *Homozygous, *HTZ *Heterozygous

### Protein modeling

**Fig. 3:**
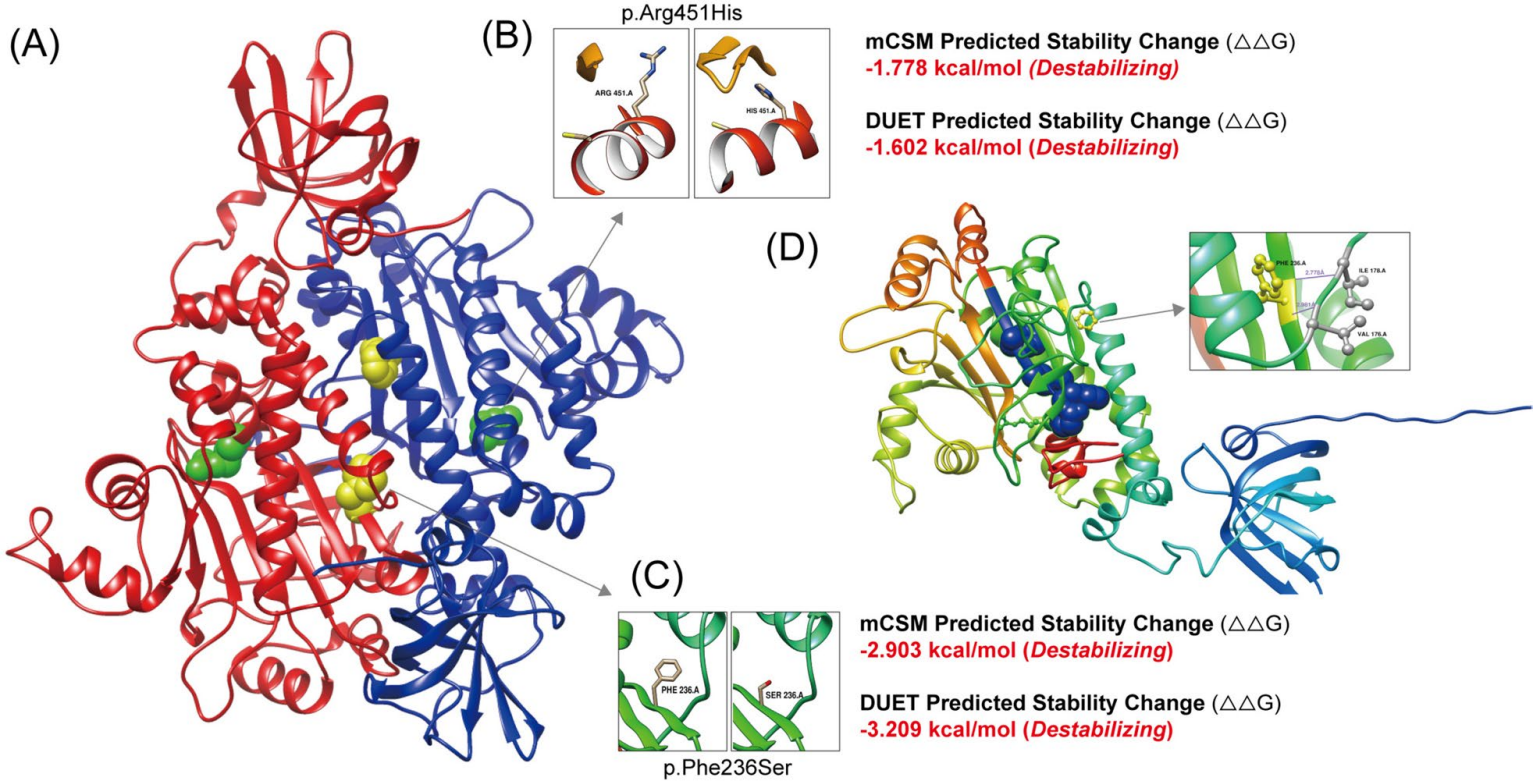
Protein modeling of NARS2. **A** The homodimer of Asn-RS in Elizabethkingia was modelled by Swiss-Model. Arg451 and Phe236 were presented by green and yellow spheres respectively. **B**, **C** The mutated residues were shown in the enlarged photos, and the predicted stability impact through mCSM and DUET was shown. **D** The variations in monomer predicted by AlphaFold were presented by yellow and green ball-stick. Motif 3 which was crucial in ATP binding was highlighted by blue spheres. The H bonds for Phe236 are shown in a partially enlarged view (purple lines) that are linked to Ile178 and Val176

To understand the molecular structures of the NARS2, comparative modeling was performed using the Swiss-Model. Due to the human Asn-RS crystallographic structure has not yet been clarified, the homology model based on Elizabethkingia sp. was used (QMEAND is Co Global 0.74) to predict and exhibited the structures of Asn-RS (Fig. 3A). All the two variants in this case were located in the aminoacyl-tRNA synthetase domain (http://pfam.xfam.org/family/PF00152) which play a crucial role in catalyzes the attachment of an amino acid to its cognate transfer RNA molecule. The variants in the dimer model were highlighted with yellow (p.Phe236Ser) and green (p.Arg451His) spheres. The changes of residues were visualization through UCSF Chimera and the stability of protein structure was predicted by mutations cut off scanning matrix (mCSM) (https://biosig.lab.uq.edu.au/mcsm/) and DUET (https://biosig.lab.uq.edu.au/duet/) (Fig. 3B, C). All the scores of mCSM method (http://biosig.unimelb.edu.au/mcsm/) and DUET server (http://biosig.unimelb.edu.au/duet/) that showed destabilizing for the residues’ changes. At the same time, the variants were highlighted in the monomer model with yellow and green as ball and stick (Fig. 3D). The ATP binding motif (motif 3) was displayed with blue spheres and the variant p.Arg451His was included indicating that the missense variant may affect the synthetase function of Asn-RS.

## Discussion

The aaRSs are a group of enzymes that facilitate the ligation of 20 amino acids to their molecular cognate tRNA [29]. Variations in aaRSs were reported leading to central nervous system (CNS) pathologies with epileptic encephalopathy, developmental delay, and intellectual disability [30]. NARS2 is a member of the class II family of aaRSs to catalyze the ligation of asparagine to tRNA molecules in the mitochondrion. The variant of *NARS2* was first reported in two siblings with myopathy and combined complex I and IV deficiency in skeletal muscle [4]. NARS2 deficiency may cause a decrease in oxygen consumption rates and electron transport chain activities in patient fibroblasts [1]. The specific cardiac dysfunction and neonatal diabetes phenotypes are supplied in *NARS2* variant individuals. On the whole, they mainly present status seizures, visual hearing disorder, and severe myopathy that was identified as the pathogenic gene of COXPD24 (OMIM:616,239).

A comprehensive review of NARS2 mutations was performed. Up to now, only 28 variants in NARS2 gene have been reported, and the exact genotype-phenotype correlation is not clear. The number of reported cases related to NARS2 deficiency has been gradually increasing [3, 5–7, 9, 26–28]. Recently, more individuals of *NARS2* variants have been reported [9, 22–25]. Data from this study was compared with 28 variants in NARS2 gene published studies. Their diagnosis, phenotype, variant type, zygote type, survival outcome, and clinical finding are summarized in Table 2. Domain structure and modeling of the known NARS2 variations in previous studies in Fig. 2D.

Epileptogenesis is commonly associated with neurodegeneration and bioenergetic defects and mitochondrial dysfunction decline of energy by dysfunction of the electron transport chain leading to apoptotic neuronal death [31]. As previous studies, neurodevelopmental disorders were the main features of NARS2 deficiency. Most of the patients with *NARS2* variants had focal, generalized, or myoclonic seizures and mitochondrial abnormalities such as combined complexes decreased and structurally abnormal [3]. In this study, a female infant with intermittent fever, status seizures, and GDD was described. GDD presented as difficulty in head control and roll over at her four-month-old. Status frequent focal subclinical or clinical seizures in the left frontotemporal were observed by long-term EEG monitor. Moreover, the brain structure abnormal was also detected in our patient with abnormal single and bilateral white matter atrophy in MRI. These clinical features were commonly in diseases with aaRSs gene mutations, including leukoencephalopathy with thalamus and brainstem involvement and high lactate (LTBL) cases with NARS2 variations, leukoencephalopathy with brainstem and spinal cord involvement, lactate elevation (LBSL) with Aspartyl-tRNA Synthetase 2 (DARS2) variations, and Alanyl-tRNA Synthetase 2 (AARS2)-related leukoencephalopathy [29]. It seems that there may be a shared mechanism of mitochondrial dysfunction in these disorders.

Severe myopathy was another characteristic clinical feature for cases with *NARS2* variant. It is well known that mitochondrial dysfunction will affect tissues request high-energy such as brain, muscle, and heart. Patients with NARS2 deficiency usually develop muscle weakness of limbs and face muscles. Myocardial dysfunction in this case was represented with CK-MB and PRO-LPBN evaluated. Heart phenotype in patients with NARS2 deficiency was rare with mitral valve prolapse [9] and cardiac dysfunction [22], while myocardial dysfunction has been reported in other aaRSs, including AARS2 [32] and Lysyl-tRNA synthetase (KARS) [7, 33]. The reported patient with cardiac dysfunction has same phenotype with our patient and persistent elevation of serum hepatic and myocardial enzymes, but further investigation is necessary to verify whether NARS2 variants lead to cardiomyopathy.

Individuals with the same variant could exhibit different phenotypes in identical [4] or unrelated [7] families. While some clinical features with vision impairment were specifically present in some cases but not found in our patient. This may be explained by tissue specificity that consistent with other mitochondrial diseases [34]. The broad phenotypic variability of NARS2 related disease present from an infantile lethal phenotype to mild non-progressive disease. Therefore, there may be no strong association between the genetic variants and disease severity [7].

All variants observed in *NARS2* were located in functional domains of NARS2 (Fig. 1D). Most of them were missense and may lead to protein dysfunction by changing the stability or interactions with other biological molecules [35]. The compound heterozygous variations of our patient in *NARS2* [c.1352G > A (p.Arg451His); c.707T > C (p.Phe236Ser)] are located in the aminoacyl-tRNA synthetase domain. This domain contains three conservative motifs which are also found in other classII aaRSs. Among them, motif 3 contains strictly conserved arginine (Arg) residue that plays a crucial role in adenosine triphosphate (ATP) binding function [36]. Based on protein modeling analyses, the variant c.1352G > A; p.Arg451His (Fig. 3) changes Arg to His that is from a conserved non-aromatic to an aromatic, differently shaped, and this changing conserved Arg in motif 3 may affect ATP binding and the NARS2 function. Furthermore, another pathogenic mutation for the change of the Arg residue (from Arg to Cys) was also shown in another patient with Leigh syndrome [20]. Another variant in our patient in the 236th amino acid changes one amino acid to another that is more polar, smaller, and more flexible. It was found to have intermolecular hydrogen bonds with the 176th and 178th residues that were contained in conserved motif 1 (Fig. 3D). The crucial role of motif 1 [37] in dimerization may be affected by Phe236Ser. Meanwhile, another changed residue in 236th (from Phe to Cys) was found in patients with the infantile-onset neurodegenerative disorder [5] which explains the pathogenicity of this variation. The two NARS2 variants in our patient were predicted by mCSM and DUET software to have a stability change in the structure of the protein (Fig. 3). Unfortunately, our study was lacking in the validation of in vivo or vitro experiments. Given the patient’s ultimate demise, we will address this shortcoming in our future research.

In conclusion, we identified the novel compound heterozygous variants in an infantile-onset patient with status epilepticus and neurodegenerative disorder with final diagnosis as mitochondrial encephalomyopathy. Our study expands the genotype spectrum of COXPD24 and highlights the critical role of NARS2 in epilepsy and neurodevelopment.

### Supplementary Information

**Additional file 1: Supplementary Table 1. **The candidate pathogenic genes variants in our patient.

## Data Availability

The DNA sequence data and Genetic variation data were used in our study. The data that support the findings of this study are available from the corresponding author upon reasonable request. Supplementary data to this article can be found online at https://www.ncbi.nlm.nih.gov/clinvar/variation/2663835/?oq=SCV004171024&m=NM_024678.6(NARS2):c.707T%3EC%20(p.Phe236Ser), https://www.ncbi.nlm.nih.gov/clinvar/variation/2663834/?oq=SCV004171023&m=NM_024678.6(NARS2):c.1352G%3EA%20(p.Arg451His).

## Abbreviations

GDD: The global developmental delay
NGS: Next-generation sequencing
COXPD24: combined oxidative phosphorylation deficiency 24
Asn-RS: Asparaginyl-Trna synthetase
aaRSs: Aminoacyl-tRNA synthetases
WES: Whole exome sequencing
BWA: Burrows-Wheeler Aligner
ACMG: American College of Medical Genetics and Genomics
gnomAD: Genome aggregation database
AR: Autosomal recessive
PCR: Polymerase chain reaction
EEG: Electroencephalograph
MRI: Magnetic Resonance Imaging
CK-MB: Myocardial creatine kinase
PRO-LPBN: B-type natriuretic peptide precursor
BAEP: Brainstem auditory evoked potentials
MAF: Minor allele frequency
mCSM: Mutations cut off scanning matrix
CNS: Central nervous system
LTBL: Leukoencephalopathy with thalamus and brainstem involvement and high lactate
LBSL: Leukoencephalopathy with brainstem and spinal cord involvement lactate elevation
DARS2: Aspartyl-tRNA Synthetase 2
AARS2: Alanyl-tRNA Synthetase 2
KARS: Lysyl-tRNA synthetase
Arg: Arginine
ATP: Adenosine triphosphate
